# Effects of Parent-Implemented Interventions on Outcomes of Children with Autism: A Meta-Analysis

**DOI:** 10.1007/s10803-022-05688-8

**Published:** 2022-08-22

**Authors:** Wai Man Cheng, Timothy B. Smith, Marshall Butler, Tina M. Taylor, Devan Clayton

**Affiliations:** Department of Counseling Psychology and Special Education, Brigham Young University Provo, 340 McKay Building, Provo, UT 84602 USA

**Keywords:** Parent-mediated interventions, Home-based services, Family delivered services, Parent training, Autism spectrum disorder, Meta-analysis

## Abstract

Children with autism spectrum disorder (ASD) have been shown to benefit from parent-implemented interventions (PIIs). This meta-analysis improved on prior reviews of PIIs by evaluating RCTs and multiple potential moderators, including indicators of research quality. Fifty-one effect sizes averaged moderately strong overall benefits of PIIs (*g* = 0.553), with studies having lower risk of research bias yielding lower estimates (*g* = 0.47). Parent and observer ratings yielded similar averaged estimates for positive behavior/social skills (*g* = 0.603), language/communication (*g* = 0.545), maladaptive behavior (*g* = 0.519), and to a lesser extent, adaptive behavior/life skills (*g* = 0.239). No other study, intervention, or participant characteristic moderated outcomes. PIIs with children with ASD tend to be effective across a variety of circumstances.

**Supplementary Information:**

The online version contains supplementary material available at 10.1007/s10803-022-05688-8.

Parents and professionals can begin to recognize characteristics of autism spectrum disorder (ASD) in children as young as the first year of life (e.g., Tanner & Dounavi, 2020). To facilitate developmental outcomes in cognition, adaptive behaviors, and communication, researchers recommend early identification of and support for ASD (Koegel et al., 2014; Nahmias et al., 2019; Reichow, 2011; Sandbank et al., 2020; Warren et al., 2011). Clinical recommendations are often for intensive services (e.g., 25–40 h per week, one-to-one adult to child ratio, for a year or longer), which is difficult for many families to achieve. Obtaining intensive intervention for children with ASD can prove challenging for many parents due to costs, limitations of time, travel distance, access to appropriate services, long waitlists, and lack of insurance coverage (Buescher et al., 2014; Nevill et al., 2016; Symon, 2001). Recognizing the struggles and limitations faced by families with children with ASD, professionals increasingly evaluate alternatives to service delivery (Cidav et al., 2017; Hatcher & Page, 2020; Lee et al., 2018; Meadan et al., 2016), including interventions delivered by the parents (Pi et al., 2021).

Parent-implemented interventions (PIIs; also referred to as parent training or parent-mediated interventions) entail intensive professional training to inform and support parents to assist children with developmental disabilities (e.g., Akamoglu & Meadan, 2018). PIIs promote parent–child engagement and address behavior support, communication and social interaction, and daily living skills by increasing parent skills and knowledge through didactic instruction, role play, coaching/supervision, in-home practice assignments, handouts, and/or in-person or virtual home visits (e.g., Bearss et al., 2013; Kasari et al., 2014). Although PIIs can function as the primary intervention provided, such as when professional services are difficult to access, PIIs compliment professional programs by generalizing to daily routines and by expanding the frequency and scope of interventions provided (Bearss et al., 2015a; Dawson-Squibb et al., 2020). PIIs can also increase parents’ engagement and feelings of competence (Deb et al., 2020; Jhuo & Chu, 2022; Liu et al., 2020) and decrease parental stress and strain (Iadarola et al., 2018). Examples of PIIs include adapting the Early Start Denver Model (ESDM; Dawson et al., 2010; Jhuo & Chu, 2022; Rogers et al., 2012) and Project ImPACT (Ingersoll & Dvortcsak, 2019).

Prior reviews and meta-analyses have reported positive effects of PIIs on child behaviors and communication (Black & Therrien, 2018; Dyches et al., 2018; Nevill et al., 2016; Postorino et al., 2017). For example, Deb and colleagues (2020) found mild to moderate benefits of three specific PIIs across 15 of 17 papers reviewed. Summarizing 21 PIIs in Asia, Liu et al. (2020) reported strong benefits, averaging *d* = 0.76, across a variety of child outcomes. Nevertheless, the results vary across studies (e.g., Alquraini et al., 2018; Jocelyn et al., 1998; Matthews et al., 2018; Reitzel et al., 2013). Child outcomes appear to differ based on study and PII characteristics, with most research focused on a few manualized PIIs that meet evidence-based practice criteria (Dawson-Squibb et al., 2020; Steinbrenner et al., 2020), such as Stepping Stone/Triple P (Turner et al., 2010). Comparing outcomes across PIIs will help to clarify the effectiveness of specific programs and will also clarify the circumstances when PIIs are most and least effective (across child, parent, and study characteristics).

Many scholars indicate that conclusions about the effectiveness of PIIs require improved quality of research evidence, specifically randomized experimental designs (e.g., Beaudoin et al., 2014; Dawson-Squibb et al., 2020; Oono et al., 2013), but several methodological limitations have characterized prior meta-analytic reviews of child outcomes following PIIs. First, many prior reviews have included retrospective or quasi-experimental designs that are susceptible to research bias, particularly selection bias when motivated parents seek out the intervention condition of the study. Second, previous meta-analyses have not evaluated if the results differ as a function of plausible study bias from participant attrition without intent-to-treat data, baseline differences across groups, or failing to conceal participant allocation or group conditions. Third, reviews have not distinguished outcomes based on professional observations versus parent-reports, which can provide holistic/continuous evaluations not possible by trained observers but which can vary in terms of reliability across participants/conditions and which may bias study findings (Bennetts et al., 2016). In outcome research, parents in the intervention group can presume that researchers expect child improvement, such that parents may rate their child’s outcomes differently than an independent observer blind to study conditions. It is therefore necessary to compare parent ratings with evaluators’ ratings to obtain more objective conclusions regarding PIIs' effectiveness. Fourth, studies included in prior meta-analyses have combined results across different types of conditions: waitlist controls, emotional support for parents without instruction about how to intervene with their child, or professionally delivered services without parent involvement. These differences in study conditions should be evaluated. Fifth, many prior reviews have not evaluated reports of treatment fidelity, such that differences in outcomes may be attributable to variability of treatment implementation. Sixth, some prior meta-analyses have included limited sources due to restricted search terms, which procedure may fail to locate studies using synonymous but distinct terminology. Seventh, some prior reviews have included only published articles, which may exacerbate the effects of publication bias (Van Aert et al., 2019) to overrepresent findings supportive of PIIs.

To address these multiple limitations, we conducted a meta-analytic review of randomized controlled trials (RCTs) using comprehensive search terms and extensive coding of study characteristics to obtain data beyond that of prior meta-analyses. We limited our review to group evaluations of PIIs to align with the common practice in the field of providing training to multiple parents simultaneously. Although rigorous single-subject research clearly contributes to the literature, individual parent training may yield different results that deserve separate consideration. Moreover, the different effect size calculations and circumstances in single-subject research would merit separate review. In this meta-analysis specific to RCTs, we sought to address the following research questions:

1. To what extent do parent-implemented interventions facilitate behavioral and language improvements among children with ASD compared with professional treatment, treatment as usual, or waitlist conditions?
2. To what extent do study characteristics (e.g., type of control group, source of outcome data, type of child outcome), intervention characteristics (e.g., duration and number of sessions), and participant characteristics (e.g., age, gender) moderate the effectiveness of PIIs?

## Method

Procedures aligned with the Preferred Reporting Items for Systematic Reviews and Meta-Analyses (PRISMA) Statement (Moher et al., 2009), except we do not report individual study risk of research bias. We report risk of bias summary statistics and performed analyses to investigate the possible impact of risk of bias on summary estimates of the effect (Shea et al., 2017). All review methods were established prior to conducting the review.

### Manuscript Search

We used multiple strategies to locate RCTs evaluating interventions involving parents as interventionists for their children with ASD. We searched eight electronic databases for articles entered from 1990 until June 3, 2020: Academic Search Ultimate, CINAHL, ERIC, Embase, Medline, ProQuest Dissertations, PsychINFO, and Social Sciences Abstracts. To capture relevant studies, our searches used lists of synonymous search terms and keywords that we identified using database search tools (full search terms available online supplementary material). To reduce inadvertent omissions, we searched each electronic database twice.

Additionally, we identified the following meta-analyses on related topics and searched their references for studies that met our inclusion criteria: Black and Therrien (2018), Dyches et al. (2018), Ferguson et al. (2019), McConachie and Diggle (2007), Nevill et al. (2016), Parsons et al. (2017), Postorino et al. (2017), Oono et al. (2013), Ratliff-Black and Therrien (2020), Tachibana et al. (2017), Tarver et al. (2019), and Virues-Ortega et al. (2013). As a final step, we examined the reference lists of all studies that met our inclusion criteria. To systematize our search and screening procedures, all located studies were uploaded to Covidence Systematic Review software (SaaS Enterprise, 2014).

### Inclusion Criteria and Screening

We sought RCTs involving children with ASD receiving a PII compared with professional treatment, treatment as usual, or waitlist controls on one or more of the following outcomes: expected behaviors/social skills, maladaptive behaviors, language/communication, and/or adaptive behaviors/life skills. We excluded case studies, qualitative research, quasi-experimental designs, single-subject designs, and studies that only compared different intensities/modes of the same program. Furthermore, we excluded studies in which the child participants had not been evaluated for ASD, the parents’ involvement in the intervention was unclear or consisted solely of passive reception of information, or the intervention groups delivered only medication, software/apps, music, or sleep/diet modifications. To reduce the likelihood of publication bias adversely impacting the results, we included both published (journal articles and book chapters) and unpublished studies (conference presentations, dissertations, and theses).

The screening procedure included three stages: (a) Covidence removed duplicate studies retrieved from different databases, (b) at least two independent reviewers screened articles based on title and abstract, and (c) at least two independent reviewers read full-text articles that had appeared to meet inclusion criteria. Thus, all studies located were screened twice during the initial screening and again during full-text review by individuals unaware of others’ decisions. Data managed in Covidence software indicated 91.7% inter-rater agreement for the independent initial screening based on titles and abstracts (Cohen’s *kappa* = 0.51) and 92.5% inter-rater agreement for the independent screening based on full-text review (Cohen’s *kappa* = 0.81). The first and second authors made final decisions for unresolved discrepancies and merged different publications when they reported the same data.

### Data Coding

Teams of two members each were trained for and conducted data coding. A codebook describing all study variables facilitated consistency across coders. Two independent teams coded each study, including (a) number of child participants and their gender and average age; (b) caregiver who delivered the intervention; (c) type and dosage of the intervention provided and reports of treatment fidelity; (d) comparison group type and measurement type, including parent rating or professional observation; (e) intervention effectiveness in improving parent intervention skills; (f) indicators of study bias, including baseline differences after randomization, participant attrition, intent-to-treat analyses, allocation concealment, blinding of interventionists to group conditions, and blinding of assessors to group conditions; and (g) effect size calculated using data provided in the manuscript. Data managed in Covidence software indicated moderate but acceptable reliability across the categorical variables coded (Cohen’s *kappa* = 0.43) and good reliability across the continuous variables coded (intra-class correlation coefficient = 0.85). When discrepancies occurred, coding teams met to resolve the disagreement by further investigation of the manuscript to reach a consensus.

### Computation of Effect Size Estimates

Different statistical values (e.g., *f*-tests, means and standard deviations, and *p*-values) reported in studies were initially converted to the standard metric of Cohen’s *d* using the Meta-Analysis Calculator (Wilson, 2021). Since several studies had low numbers of participants, we converted all effect sizes to Hedge’s *g* values to reduce bias associated with differences in variance that can occur in small studies (Marfo & Okyere, 2019). Where analyses were reported as statistically significant with no statistic provided, the corresponding alpha level determined the *g* value (assuming two-tailed alpha = 0.05 unless noted otherwise). Effect size *g* = 0 was set for analyses that report non-significant results with no additional information. These procedures yielded conservative effect size estimates. The direction of all effect sizes was coded uniformly: positive values indicate improved child outcomes as a function of the intervention, and negative values indicate a deleterious effect relative to the comparison group.

When studies reported multiple measures of child functioning at both pre-test and post-test, we extracted every effect size that was conceptually aligned with the description of the intervention, and we coded for differences across types of outcomes (e.g., separating effect sizes based on evaluations of child language/communication scores from those based on child adaptive behavior scores). We avoided coding effect sizes based solely on broad screeners or comprehensive diagnostic batteries inappropriately used as outcome measures. To avoid violating the assumption of statistical independence we averaged multiple effect sizes within studies, weighted by standard error or the number of participants evaluated, such that our omnibus analysis included only one effect size per study.

### Risk of Research Bias and Publication Bias

We sought to obtain high-quality research evidence by coding only studies in which participants had been randomized to treatment and control conditions, and we also evaluated the risk of bias of each article in terms of: researcher awareness of participant allocation, interventionist awareness of study conditions, outcome evaluations conducted by either parents, researchers, or observers unaware of the treatment/control group, participant baseline differences across groups, participant attrition, and intent-to-treat analyses.

The direction and statistical significance of the results impact the publication of studies, which can introduce publication bias (Van Aert et al., 2019). In a meta-analysis, publication bias can lead to overestimation of effect sizes and underestimation of false-positive results. We used multiple methods to evaluate possible publication bias: Egger’s regression test, Begg’s regression test, trim and fill analysis, and funnel plot analysis to detect distribution asymmetry.

### Statistical Analyses

We used IBM® SPSS® Statistics (Version 27.0) and STATA (Version 17.0) to analyze the data. The pooled effect size was calculated using an inverse-variance weighted random effects model. To assess the heterogeneity of the pooled effect size, we obtained the I-square (*I*^2^), tau-square (*τ*^*2*^), and Cochran’s *Q*. We specified a priori that if the omnibus effect size was heterogenous, we would conduct random effects weighted moderator analyses consisting of subgroup analyses for categorical variables and meta-regression for continuous variables. Analyses were pre-specified and conducted as planned, except for the addition of a Galbraith plot which was included after the first round of journal review to depict effect size heterogeneity and evaluate potential outliers.

## Results

### Study Selection

**Fig.1:**
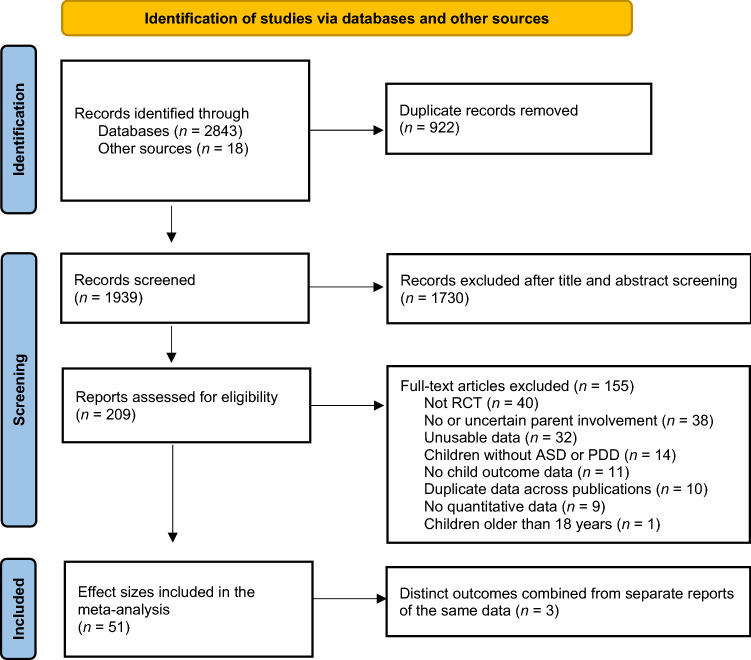
PRISMA Flow diagram of included studies

Our search strategies located 1939 non-duplicate studies which we reviewed for inclusion. After initial screening, 209 records remained for full-text review. A summary of the screening process and reasons for exclusion are reported in the PRISMA flow diagram (Fig. 1). Studies retained for coding included 3 unpublished and 51 published articles (54 total). However, three pairs of studies contained distinct but overlapping data (e.g., different measures reported in different articles with the same participants), so those studies were combined to yield one aggregate effect size each, for a total of 51 independent effect sizes.

### Descriptive Characteristics of Participants and Interventions

The 51 extracted effect sizes involved a total of 2,895 child participants with an average age of 5.5 years and an average of 17% females and 83% males. A total of 43 records (84%) involved both mother and father, 6 (12%) involved only the mother, and 2 (4%) involved parents and other caregivers. Twelve studies targeted improvements in social skills and other pro-social behaviors, 8 targeted problem behaviors, 6 targeted language/communication, and 25 were mixed, with most of those mixed studies addressing both social/positive behaviors and language/communication.

Across interventions, parents received an average of 89.6 min and 13.3 sessions of training that typically occurred weekly (73%). Child outcomes were measured using direct observations by professionals in 20 (39%) studies, parent-completed standardized instruments in 15 (29%) studies, child-completed standardized instrument in 1 study (2%), with 14 studies (28%) using multiple measures (e.g., parent and observer ratings) and 1 (2%) not providing details regarding measurement source.

Baseline scores were equivalent between children in intervention and control groups (indicating that randomization resulted in balanced participants) in 38 studies (74.5%), with 3 (5.9%) having differences that favored the intervention group, 6 (11.8%) having group differences that favored the control group, and 4 (7.8%) not reporting. Only two records (4%) had all interventionists, observers, and data analysts who were “blind” or unaware of participant allocation, 23 (45%) involved blind observers for the outcome evaluations, 17 (33%) records did not provide enough information or stated that researchers were not blind, 7 (14%) reported that both the interventionists and the outcome observers were blind, 1 (2%) only kept the interventionists unaware, and 1 (2%) kept both the outcome observer and data analysts unaware. No information was provided for allocation concealment in 17 (33%) records; 29 (57%) indicated that researchers were not involved in the randomization process, and 5 (10%) declared that researchers conducted the randomization and were aware.

Studies differed in terms of improving parents’ self-reported abilities to intervene with the children. Twenty-one studies (57%) reported that parents in the intervention group gained statistically significantly more child intervention skills than those in the control group, but 8 (17%) reported no gains or worse performance than the control group, with 22 (43%) not reporting data regarding changes in parents’ intervention skills as a function of the intervention. Treatment fidelity was not measured or reported in 20 (39%) of the records; 18 (35%) reported that the PII was implemented as intended; 12 (23%) assessed fidelity but did not report if the intervention was implemented as intended; and 1 (2%) reported low treatment fidelity. The average overall participant attrition across studies was very low, averaging 2.89 participants (9.7%) in the experimental group and 2.34 participants (8.4%) in the control group.

Studies used different types of comparison groups, with only one study (2%) comparing PIIs to a specific intervention conducted by professionals without parent involvement, 17 (33%) using unspecified “treatment as usual” conducted by professionals, 7 (14%) involving parent support but not an active PII, and 26 (51%) using waitlist control groups. There were 35 (69%) outcomes based on data from intervention completers and 16 (31%) based on intent-to-treat analyses. A summary of the characteristics of the included studies appears in Table 1.

**Table 1:** Overview of 54 studies yielding 51 independent effect sizes

Author(s)	Country	N	Mean age (Years)	Effect size (*g*)	Intervention name	Relevant dependent variables
Aldred et al., 2004	United Kingdom	28	3.83	0.25	Social communication intervention	PB, AB, L
Alquraini et al., 2018	Saudi Arabia	28	3.70	2.44	Responsive teaching	PB, L
Alvarado, 2017	United States	30	3.73	1.31	Sensoriaffective interactional attunement scale-guided intervention	PB
Amrollahi far, 2017	Iran	30	7.23	0.51	Play therapy training	L
Bearss et al., 2015b, 2016	United States	180	4.75	0.36	Behavioral interventions	MB
Beaudoin et al., 2019	Canada	19	2.13	0.00	Parent implemented early start denver model (P-ESDM)	PB
Brian et al., 2017	Canada	62	2.10	0.99	Social ABCs parent-mediated intervention	PB, L
Byford et al., 2015	United Kingdom	146	4.00	0.41	Pre-School Autism Communication Trial (PACT)	L
Carter et al., 2011	United States	50	1.67	0.00	Hanen’s ‘More Than Words’	PB, L
Casenhiser et al., 2013, 2015	Canada	51	3.71	0.72	Milton & ethel harris research initiative (MEHRI)	PB, L
Cook et al., 2019	Australia	31	5.50	0.00	Cognitive behavioral therapy (CBT)	MB
Dekker et al., 2019	Netherlands	69	11.00	0.43	Social skills group training	PB
Frankel et al., 2010	United States	68	8.53	0.22	Children’s friendship training	PB, MB
Ginn et al., 2017 and Clionsky, 2012 (same data)	United States	30	4.72	0.60	Child-directed interaction training	PB, MB
Green et al., 2010	United Kingdom	152	3.75	0.17	PACT	PB, L
Handen et al., 2013	United States	124	7.43	0.20	Research units on pediatric psychopharmacology —autism network (RUPP)	MB
Handen et al., 2015	United States	64	7.95	0.52	RUPP	MB
Hardan et al., 2015	United States	47	4.10	0.41	Pivotal response treatment (PRT)	L
Ho & Lin, 2020	Taiwan	24	4.04	0.49	Developmental individual-difference relationship-based model	PB, AB, L
Iadarola et al., 2018	United States	180	4.75	0.77	RUPP	MB
Jocelyn et al., 1998	Canada	35	3.60	-0.03	Autism preschool program	PB, AB, L
Kasari et al., 2010	United States	38	2.57	0.62	Joint attention intervention	PB
Kuravackel et al., 2018	United States	33	8.08	0.69	Collaborative model for promoting competence and success for hope	MB
Lehtonen et al., 2020	Finland	20	4.13	0.31	Parent-led eye contact-specific training	PB, L
Lindgren et al., 2020	United States	38	4.35	1.54	Functional communication training	MB
Matthews et al., 2018	United States	22	15.27	1.80	Peers-mediated model of program for the education and enrichment of relational skills (PEERS®)	PB
McDaniel et al., 2020	United States	40	4.03	0.49	PRT	L
Nowell et al., 2019	United States	17	6.82	0.81	Growing, Learning, and Living with Autism (GoriLLA)	PB
Pajareya & Nopmaneejumruslers, 2011	Thailand	32	4.50	1.05	Developmental, Individual-Difference, Relationship-Based (DIR)/Floortime™	PB
Pashazadeh Azari et al., 2019	Iran	33	6.82	0.39	Contextual interventions for ASD	AB
Rahman et al., 2016	India & Pakistan	59	5.43	0.60	PACT	L
Reitzel et al., 2013	Canada	13	4.88	-0.03	Functional behavior skills training	MB, AB
Roberts et al., 2011	Australia	57	3.55	0.61	Building blocks program	PB, MB, L
Schertz et al., 2013	United States	23	2.18	0.48	Joint attention mediated learning (JAML)	L
Schertz et al., 2018	United States	131	2.06	0.48	JAML	PB
Scudder et al., 2019	United States	19	5.62	0.48	Parent–Child Interaction Therapy (PCIT)	PB, MB
Shire et al., 2016	United States	83	2.58	0.80	Joint Attention, Symbolic Play, Engagement, and Regulation (JASPER)	PB
Shum et al., 2019	Hong Kong	66	13.51	0.56	PEERS®	PB, MB
Siller et al., 2013	United States	70	4.76	0.17	Focused playtime intervention	L
Sofronoff et al., 2004	Australia	100	9.33	1.08	Comic strip conversations and social stories	PB, MB
Sofronoff et al., 2007	Australia	45	10.78	1.11	CBT	MB
Solomon et al., 2008	United States	19	8.15	0.53	PCIT	PB, MB
Solomon et al., 2014 and Mahoney & Solomon, 2016	United States	128	4.18	0.36	Play and Language for Autistic Youngsters (PLAY) project home consultation model	PB, L
Tellegen & Sanders, 2014	Australia	64	5.67	0.47	Stepping Stones Triple P (SSTP)	MB
Tonge et al., 2014	Australia	70	4.00	0.75	Parent Education and Counselling (PEAC), Parent Education and Behavioral Management (PEBM)	All
Turner-Brown et al., 2019	United States	49	2.47	0.47	Family Implemented TEACCH for Toddlers (FITT)	PB, L
Valeri et al., 2019	Italy	34	4.30	0.57	Cooperative Parent-Mediated Therapy (CPMT)	PB
Whittingham et al., 2009	Australia	59	5.91	0.21	SSTP	MB
Wong & Kwan, 2010	Hong Kong	17	2.21	0.00	Autism-1-2-3	PB, L
Yoo et al., 2014	Korea	47	13.78	1.15	PEERS®	PB
Zand et al., 2018	United States	21	5.84	0.98	Positive parenting program	MB

*PB* positive behavior/social skills, *MB* maladaptive behavior, *AB* adaptive behavior/life skills, *L* language/communication

### Omnibus Analysis

**Fig. 2:**
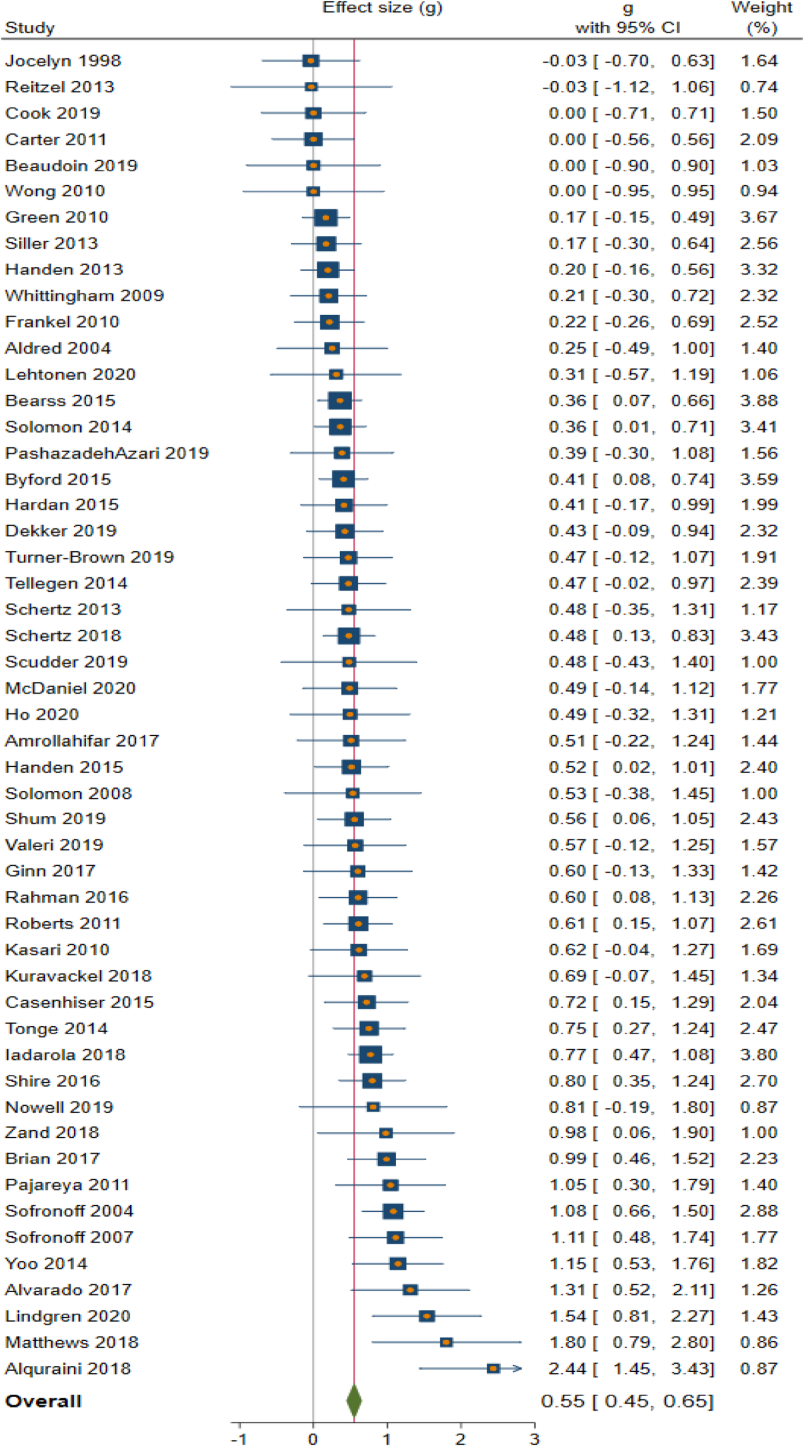
Forest plot of effect size and 95% confidence interval of RCTs in the meta-analysis

Analyses were conducted using Hedges’ *g* to minimize the likelihood of small study bias. Across all 51 effect sizes, the random effects weighted average was *g* = 0.55 (95% confidence interval *g* = 0.45 to 0.65, *p* < 0.0001; see Fig. 2).

**Fig. 3:**
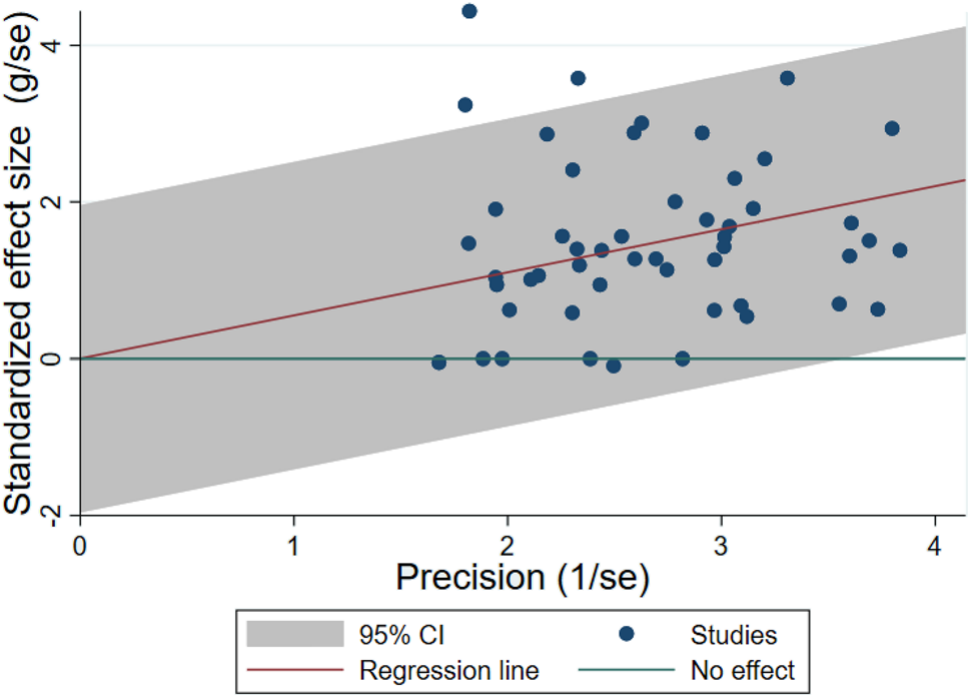
Galbraith Plot of 51 effect sizes from RCTs of parent-implemented interventions

Effect sizes ranged from *g* = − 0.03 to 2.44, with small but statistically significant heterogeneity (*I*^*2*^ = 37.6%; *τ*^*2*^ = 0.05; *Q* = 86.4, *p* < 0.001). Three of 51 effect sizes fell outside the expected distribution in a Galbraith plot (Fig. 3), but that percentage (5.9%) approximated what would have been found by chance 95% of the time. Thus the data exhibited reasonable levels of variation around the mean, with 6 (12%) studies finding no effects of PIIs, 20 (39%) finding small to moderate effects, 12 (24%) finding moderate to large effects, and 13 (25%) finding large to very large effects. We evaluated possible moderating variables that may have accounted for differences in findings across studies.

### Subgroup Analyses by Child Outcome

Within studies, authors evaluated different types of child outcomes. We categorized those outcomes into the following four groups: expected behaviors/social skills, maladaptive behaviors, adaptive behavior/life skills, and language/communication. We analyzed these data separately to ascertain the degree to which different kinds of outcomes were impacted by PIIs. Across 30 studies reporting child outcomes in terms of expected behavior/social skills, the random effects weighted average was *g* = 0.603 (95% CI 0.45 to 0.75, *p* < 0.001). These results were characterized by moderate and statistically significant heterogeneity (*I*^*2*^ = 46.6, 95% CI 18 to 65; *Q* = 54.3, *p* = 0.003). These results did not differ across parent versus observer ratings of child outcomes (Table 2).

**Table 2:** Random Effects weighted effect sizes of child outcomes by rater

Outcome	Rater	*k*	Effect size (*g*)	95% CI	*p*
Expected behaviors/social skills					
	Parent	12	0.60	0.38 to 0.82	< .0001
	Observer	22	0.63	0.45 to 0.81	< .0001
Maladaptive behaviors					
	Parent	16	0.55	0.40 to 0.71	< .0001
	Observer	5	0.68	0.19 to 1.17	.006
Adaptive behavior/life skills					
	Parent	4	0.15	− 0.25 to 0.55	*ns*
	Observer	2	0.63	0.22 to 1.05	.003
Language/communication					
	Parent	5	0.32	0.06 to 0.57	.015
	Observer	16	0.55	0.32 to 0.79	< .0001

*k* = number of effect sizes specific to parent or observer ratings, with some studies reporting both parent and observer data. *g* = Hedges’ *g*, which is similar to Cohen’s *d* but which provides a bias correction for small sample sizes

Across 20 studies evaluating child outcomes in terms of maladaptive behavior, the random effects weighted average was *g* = 0.519 (95% CI 0.37 to 0.67, *p* < 0.001). These results were characterized by small but statistically significant heterogeneity (*I*^*2*^ = 37.1, 95% CI 0 to 63; *Q* = 30.2, *p* = 0.049). These results did not differ across parent versus observer ratings of child outcomes (Table 2).

Across six studies evaluating child outcomes in terms of adaptive behavior/life skills, the random effects weighted average was *g* = 0.239, with that value not reaching statistical significance (95% CI − 0.11 to 0.59, *p* > 0.05). These results were characterized by small and statistically non-significant heterogeneity (*I*^*2*^ = 31.5, 95% CI 0 to 72; *Q* = 7.3, *p* = 0.20). Averaged parent ratings of child outcomes tended to be lower than professional ratings (Table 2), but this difference was attributable to a single outlier with a value of *g* = − 0.795.

Across 19 studies evaluating child outcomes in terms of language/communication skills, the random effects weighted average was *g* = 0.545 (95% CI 0.34 to 0.75, *p* < 0.001). These results were characterized by moderate, statistically significant heterogeneity (*I*^*2*^ = 61.2, 95% CI 36 to 76; *Q* = 46.5, *p* < 0.001). Results did not differ across parent versus professional ratings of child outcomes (Table 2).

### Evaluations of Publication Bias

**Fig. 4:**
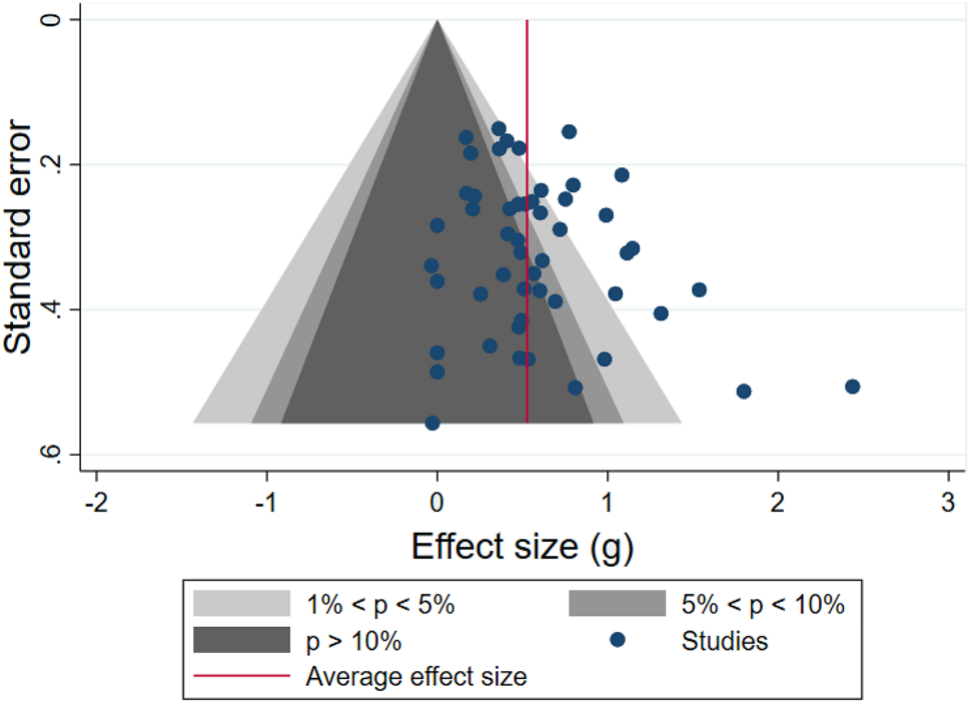
Contour-enhanced funnel Plot of 51 effect sizes from RCTs

We administered several tests to detect plausible publication bias. Both Egger’s regression test and Begg’s test did not reach statistical significance, suggesting the effect sizes were distributed normally. We also generated a contour-enhanced funnel plot to examine publication bias (see Fig. 4). In the plot, the data were only slightly asymmetrical, with most data evenly distributed around the mean. Subsequent trim-and-fill analyses did not identify any missing studies in the distribution. Therefore, publication bias did not appear to be a likely threat to the results of this meta-analysis.

### Evaluations of Moderation by Participant, Intervention, and Study Characteristics

To evaluate whether effect size heterogeneity could be explained by other variables, we performed moderator analyses of participant, intervention, and study characteristics. Participant characteristics included the gender and mean age of child participants, as well as the identity of the caregiver performing the intervention (e.g., mother, father, both parents). Intervention characteristics included the type of intervention, intervention effectiveness in improving parents’ skills, treatment fidelity, and dosage (number of sessions, frequency, and duration). Study characteristics included year of publication and comparison group type (e.g., alternative intervention, wait list controls).

None of the variables evaluating participant, intervention, or study characteristics reached statistical significance in the random effects weighted meta-regressions and subgroup ANOVA analogues that we conducted. Thus, we found no indication of effect size moderation.

### Evaluations of Risk of Study Bias

We had coded for five indicators of risk of study bias: allocation concealment, baseline differences across groups, masking of individuals involved in the interventions, participant attrition, and outcome data based on completers or intent-to-treat analyses. We observed no statistically significant differences in study effect sizes across these five variables when evaluated separately (*p* > 0.05). However, after combining indicators into one overall indicator of risk of research bias (e.g., Sterne et al., 2019), we observed a statistically significant random effects weighted correlation between study risk of bias and the corresponding effect size (*r* = 0.30, *p* = 0.02). The 29 studies with only one or two variables indicating risk of bias averaged *g* = 0.47 (95% CI 0.35 to 0.59, *p* < 0.001) while the 22 studies with three or more of the five variables indicating risk of bias averaged *g* = 0.68 (95% CI 0.51 to 0.84, *p* < 0.001). Thus, studies conducted with higher levels of research rigor yielded less robust child outcomes than studies failing to conduct or report aspects of RCT methodology.

## Discussion

### PII Effectiveness with Children with Autism Spectrum Disorder

The findings of this meta-analysis confirmed overall favorable effects of PIIs for children with ASD. Moderately strong improvements in child outcomes relative to control conditions were found (*g* = 0.55), even when restricting analyses to studies with lower risk of research bias (*g* = 0.47). The observed beneficial effects of PIIs were consistent with some prior meta-analyses and systematic reviews (Deb et al., 2020; Liu et al., 2020; Ratliff-Black & Therrien, 2020), but more favorable than data reported in others (Nevill et al., 2016; Oono et al., 2013; Tachibana et al., 2017).

The meta-analytic data indicated that PIIs helped children with ASD to improve in positive behaviors/social skills (Kent et al., 2019; Ona et al., 2019; Soares et al., 2020), maladaptive behaviors (Black & Therrien, 2018; Gerow et al., 2017; Postorino et al., 2017), and language/communication (Fuller et al., 2020; Sandbank et al., 2020; Wang et al., 2021; Yu et al., 2020). Although improvements were also made in adaptive behaviors/life skills (Rodgers et al., 2021), the averaged results tended to be much smaller (*g* = 0.24) than those obtained for the other outcome types, which ranged from *g* = 0.52 to 0.60. However, this difference may have occurred because all studies evaluating adaptive behavior/life skills had also addressed other aspects of child functioning; no interventions were exclusive to child adaptive behaviors/life skills.

Overall, PIIs enable parents to apply skills learned from professionals to real-life situations. PIIs likely benefit children with ASD more than comparison conditions because parents are continuously present with the child and can augment the effectiveness of any other intervention provided. Analyses were conducted to evaluate factors such as PII dosage that could have influenced PII outcomes.

### Outcomes Across Participant, Intervention, and Study Characteristics

In this meta-analysis, we intentionally evaluated multiple participant, intervention, and study characteristics that could have moderated the effectiveness of PIIs with children with ASD. However, none of the variables we evaluated reached statistical significance. Results tended to be similar across a wide variety of circumstances.

In terms of study participants, PIIs tended to yield equivalent results irrespective of the average child age or the percentage of female participants. No differences were observed when the mother, father, or both implemented the intervention. It is also important to note that parents’ ratings of their own children yielded equivalent results to ratings by observers. This finding suggests that, on average, parent data do not necessarily bias research findings when parents remain aware of the research hypotheses. It also suggests that professional observations, necessarily limited in duration and scope, yield similar averaged estimates to the more continuous observations of parents.

Findings tended to be similar across intervention characteristics of dosage, fidelity, and changes in parents’ abilities to assist their children. These results were unexpected and raise questions about causality, since more intensive treatments conducted with fidelity that significantly improved parent skills should result in greater child gains. However, this finding is qualified by the fact that studies often did not report adequate information, with 43% not evaluating treatment fidelity, 39% not reporting parent changes in abilities to work with their children, and 19–39% not reporting details about intervention duration or frequency, with many only reporting the expected hours per week and only three studies logging parent intervention times. Future research needs to report intervention details.

Missing data also impacted our evaluations of risk of research bias. Studies often did not report allocation concealment (33%) or masking personnel involved in the interventions (33%), with 57% not reporting intent-to-treat analyses. Notably, studies that reported multiple procedures to reduce risk of research bias tended to yield outcomes of lower magnitude (*g* = 0.47) than studies reporting fewer procedures to reduce bias (*g* = 0.68). We therefore urge scholars to include risk of bias considerations when designing and reporting their studies.

### Comparison with Previous Meta-Analyses and Systematic Reviews

Our meta-analysis included only RCTs yielded omnibus results that were very similar to the findings of prior meta-analyses that had included studies using quasi-experimental designs (e.g., Dyches et al., 2018). Notably, our review also yielded smaller inconsistency in findings across studies than prior reviews, which typically have reported higher variability (e.g., Beaudoin et al., 2014; Deb et al., 2020; Liu et al., 2020; Nevill et al., 2016; Parsons et al., 2017; Postorino et al., 2017; Ratliff-Black & Therrien, 2020; Tachibana et al., 2017; Tarver et al., 2019). Thus, restricting our focus to high quality research evidence (RCTs) apparently diminished sources of random error across studies and thus strengthened our confidence in the effectiveness of PIIs.

We explicitly sought to improve upon the methodology of prior reviews by conducting extensive literature searches and by evaluating possible indicators of study bias and effect size moderation. Our analyses of multiple indicators of research bias provide a major contribution to the field. We observed that many RCTs tended to be of low to moderate overall quality. All but two studies failed to report keeping researchers blind to participant allocation and isolated from the PII conditions/training. About 41% of studies did not report keeping researchers blind to outcome evaluations. Only 35% confirmed implementation fidelity. On a positive note, randomization tended to balance participants on baseline characteristics, as intended. A major strength of the PIIs evaluated in this meta-analysis was consistently low rates (< 10%) of participant attrition. Given high participant retention, intent-to-treat analyses should have yielded very similar outcomes to the results reported, but unfortunately only 31% of studies reported intent-to-treat analyses.

This meta-analysis also made a substantive contribution to the literature by evaluating differences in study outcomes when rated by either parents or researchers. Although parent-reported measures can be problematic for several reasons (Nevill et al., 2016; Oono et al., 2013; Wolstencroft et al., 2018), including expectancy and reporter bias (Tarver et al., 2019), the meta-analytic data suggest that such biases are not as systematic as anticipated. Parent observations have the advantages of being continuous across a broad range of circumstances, whereas professional observations are necessarily limited in duration and context. Researchers concerned about measurement bias may consider using multiple sources of data, including teacher-report measurements for child outcomes, which we rarely found in literature.

Our literature search was extensive and located studies from Japan, Korea, Thailand, Hong Kong, Taiwan, China, India, Pakistan, Iran, and Saudi Arabia. Unfortunately, many of those studies were excluded due to either not being RCTs or missing essential information (e.g., effect size data, active parent participation). Crucial information (e.g., frequency, duration, sessions, treatment fidelity) was missing in many studies, consistent with the findings and recommendations of Liu et al. (2020). Only nine studies from non-English speaking countries were included in our final analyses. Although we sought to improve on prior reviews, incomplete information hindered our ability to investigate how PIIs may work for non-Western populations with ASD. As Liu et al. (2020) emphasized, there is a need to increase research quality worldwide, particularly in developing nations.

Child gender was not a variable addressed in prior reviews. Across the studies we reviewed, females comprised an average of 16.8% of participants. When looking at individual studies' descriptive statistics, Kuravackel et al. (2018) was the only study with a high proportion of female participants with ASD (78.8%). Males with ASD were over-represented compared to females with ASD in the research we located, but consistent with the ratio of males to females with ASD. We could not find any interactions of the percentage of females with ASD with the effectiveness of PIIs. Similarly, we found no differences across child age, but the mean age of child participants was 5.5 years. Future research can consider intervention effectiveness among older children, as well as several other key considerations raised by this review.

### Limitations and Directions for Future Research

Results of this meta-analysis should be interpreted in the context of several limitations. First, studies in this meta-analysis were almost always published, with only three (5.6%) being unpublished, even though we explicitly searched for unpublished studies. Although it stands to reason that scholars who conduct RCTs are more likely to persist to publication, it is also plausible that some RCTs with non-significant findings may not have been submitted for publication. We found no evidence of publication bias using standard meta-analytic methods, but it remains possible that unpublished data evaded our comprehensive search. Second, the literature search and subsequent coding required extensive time, with personal circumstances precluding updated coding after 2020. Third, the literature was characterized by a wide variety of interventions. It was therefore surprising that the child outcomes across different PIIs did not statistically differ, with a relatively small percentage of variability in outcomes across studies. Similarly, we do not know why intervention dosage was not predictive of child outcomes in this meta-analysis, except that effect size heterogeneity was small (with little variance to explain across studies). Fourth, we did not evaluate long-term outcomes because only a few studies included extended follow-up data. Future research needs to evaluate the effectiveness of PIIs over time (Deb et al., 2020; Liu et al., 2020; Parsons et al., 2017). Fifth, although we tried to include studies outside of North America, other world regions remained under-represented. Sixth, the studies did not adequately represent female children. Most of the RCTs consisted of predominantly male samples. Finally, we did not code for some potential moderators such as socioeconomic status, parent education level, parenting style, and marital status since those variables were inconsistently reported in the literature.

In addition to addressing the considerations just listed (e.g., long-term follow-up data and cultural differences), future research will need to consider ways to improve parent access to and utilization of PIIs. PIIs may be particularly useful for families unable to afford intensive ASD services or with limited access to nearby ASD services. Researchers can also examine how parent-implemented interventions impact the intersection of family functioning and child development (Stahmer & Pellecchia, 2015). Improved rigor and reporting of essential information remain high priorities. In particular, it is essential that researchers report the effectiveness of PIIs in improving parents’ abilities to facilitate child improvement, data which were reported only 57% of the time; researchers and future reviewers must evaluate this variable since evaluations of PIIs cannot assume that parents enact the skills taught them by professionals. Lastly, although RCTs provide strong research evidence, we invite other scholars to summarize rigorous single subject and multiple baseline designs that also contribute to our understanding of PII effectiveness. A synthesis of that important work will provide additional details about PIIs that cannot be provided in RCTs that average data across participants.

## Conclusion

Data from 51 effect sizes extracted from RCTs involving 2895 children have demonstrated moderately strong benefits of PIIs compared with usual treatment and waitlist conditions. Child improvements were observed in positive behavior/social skills, maladaptive behaviors, and language/communication skills, with smaller gains in adaptive behavior/life skills. Different approaches to parent training and interventions yielded similar outcomes, whether a parent or professional provided the evaluations.

We restricted our analyses to RCTs, which reduced the likelihood of biases adversely impacting the results. Nevertheless, individual studies varied in terms of research quality. Scholars will benefit from considering the complexities of studying parent influence on children and should explicitly report attempts to reduce risk of research bias. When accounting for risk of research bias, the average estimate of PII effectiveness diminished slightly from *g* = 0.53 to g = 0.47, which remains a moderately strong improvement over treatment as usual and waitlist conditions.

A large body of data now supports services provided by parents and caregivers. Educational and human services and relevant public policy initiatives have an empirical basis for promoting PIIs, particularly when professional services cannot easily be accessed by all children in need of services.

### Supplementary Information

Below is the link to the electronic supplementary material.

Supplementary file1 (DOCX 16 KB)
