# Supplementary material for: Effects of Parent-Implemented Interventions on Outcomes of Children with Autism: A Meta-Analysis
Source: J Autism Dev Disord. 2022 Aug 22;53(11):4147–63. doi: 10.1007/s10803-022-05688-8 (PMC10539413; doi:10.1007/s10803-022-05688-8)
Supplement: Supplementary file 1 — Supplementary file1 (DOCX 16 KB) [file 10803_2022_5688_MOESM1_ESM.docx]

**Search Strings for a Meta-analysis of Parent-Implemented Interventions**

**Title: “Effects of Parent-Implemented Interventions on Outcomes of Children with Autism: A Meta-Analysis” submitted to the *Journal of Autism and Developmental Disorders***

**EMBASE search terms**

***NOTE: If you copy and paste these terms into Embase and find an error, open this document in Google Docs and then try copying and pasting again. There seemed to be an issue with copying text from the preview document (instead of the opened document).***

('child parent relation'/exp OR 'child parent relation' OR 'child parent relationship'/exp OR 'child parent relationship' OR 'parent child relation'/exp OR 'parent child relation' OR 'parent child relationship'/exp OR 'parent child relationship' OR 'parent-child relations'/exp OR 'parent-child relations' OR 'parental role'/exp OR 'parental role' OR 'parenting'/exp OR 'parenting' OR 'parent of' OR 'mother* of' OR 'father* of' OR 'family with' OR 'families with' OR 'child rearing'/exp OR 'child rearing' OR 'mother* with' OR 'father* with' OR 'rear* child*' OR 'mother'/exp OR 'mother' OR 'motherhood'/exp OR 'motherhood' OR 'mothering'/exp OR 'mothering' OR 'mothers'/exp OR 'mothers' OR 'father'/exp OR 'father' OR 'fatherhood'/exp OR 'fatherhood' OR 'fathers'/exp OR 'fathers' OR parent* OR 'caregiver'/exp OR 'care giver' OR 'caregiver* of' OR 'carer*' OR 'family caregiver' OR 'family caregivers' or 'family-centered’ or ‘family delivered’ or ‘family-delivered’ or 'family centered care')

AND

('child'/exp OR child OR child* OR 'adolescent'/exp OR adolescent OR adolescen* OR 'teenager'/exp OR teen* OR 'youth'/exp OR youth OR 'juvenile'/exp OR juvenile* or 'under 18' or preschool or school-age*)

AND

(autis* OR asperger* OR 'asd'/exp OR asd OR 'autism'/exp OR autism OR 'asperger syndrome'/exp OR 'asperger syndrome')

AND

('intervention study'/exp OR 'early childhood intervention'/exp OR 'early childhood intervention' OR 'early education intervention' OR 'early educational intervention' OR 'early intervention (education)' OR 'early intervention, educational' OR 'education early intervention' OR 'educational early intervention' OR 'home care'/exp OR 'domestic health care' OR 'domiciliary care' OR 'home treatment' OR 'home care' OR 'programmed instruction' OR 'programmed teaching' OR 'remedial teaching' OR 'teaching material' OR 'teaching materials' OR 'teaching method' OR 'teaching program' OR 'teaching programme' OR 'teaching, programmed' OR 'intervention'/exp OR intervention* OR 'treatment'/exp OR treatment OR 'clinics'/exp OR clinics OR 'parent training'/exp OR 'parent training' OR 'program evaluation'/exp OR 'program evaluation' OR 'educational programs' OR 'parent workshop' OR 'parent involvement' OR 'parents as teachers' OR 'home instruction' OR 'home intervention' OR 'home visits'/exp OR 'home visits' OR 'home program*' OR 'family program*' OR 'family delivered' OR 'parent delivered' OR therap* OR 'therapy'/exp OR therapy OR session* OR 'program*'/exp OR 'parent mediated' OR 'parent program*')

AND

([controlled clinical trial]/lim OR [randomized controlled trial]/lim)

NOT (intestin* or chromatographic or oxytocin or vitamin* or 'fatty acid' or protein* or inflammation or inflammasome or 'gene variants' or acetylcysteine or chorioamnionitis or mice or mouse or 'rat' or 'rodent' or pyrin or interleukins)

**Academic search ultimate/Medline/Social work abstract/CINAHL**

(Parenting or "parent* of" or "carer of" or "mother* of" or "father* of" or "caregiver* of" or "famil* with" or "child rear*" or "mother* with" or "father* with" or "rear* child*" or family-centered)

AND

(child* or adolescen* or teen* or youth or “under 18” or juvenile* or preschool* or school-age*)

AND

(Autis* or Asperger* or ASD)

AND

(random* or "controlled trial" or "clinical trial" or experiment*)

AND

(SU intervention or SU treatment or SU clinic or SU "parent training" or SU "parent program*" or SU "parent mediated" or SU "parent delivered" or SU “family delivered” or SU "parent-delivered" or SU “family-delivered” or SU counseling or SU psychoeducation or SU "program* evaluation" or "educational program*" or SU "parent workshop" or SU "parent involvement" or SU "parents as teachers" or SU "home instruction" or SU "home intervention" or SU "home visits" or SU "home program*" or SU "family program*" or SU program* or SU service* or counseling or therap* or session* or “training for parent*” or “training of parent*” or “parent* home interven*” or  “parent-mediated” or “parent mediated” or “parent* program*” or “parent* interven*” or “interven* with parent*” or “intervent* for parent*”)

NOT

(intestine or chromatographic or oxytocin or proteins or inflammation or inflammasome or "gene variants" or acetylcysteine or chorioamnionitis or mice or mouse or rat or pyrin or interleukins)

**ERIC/APA PsycInfo Search**

(Parenting or "parent* of" or "carer of" or "mother* of" or "father* of" or "caregiver* of" or "famil* with" or "child rear*" or "mother* with" or "father* with" or "rear* child*" or caregiver* or family-centered)

AND

(child* or adolescen* or teen* or youth or “under 18” or juvenile* or preschool* or school-age*)

AND

(Autis* or Asperger* or ASD)

AND

(random* or "controlled trial" or "clinical trial" or experiment*)

AND

(DE intervention or DE treatment or DE clinic or DE "parent training" or DE "parent program*" or DE counseling or DE psychoeducation or DE "program evaluation" or "educational programs" or DE "parent workshop" or DE "parent involvement" or DE "parents as teachers" or DE "home instruction" or DE "home intervention" or DE "home visits" or DE "home programs" or DE "family programs" or DE counseling or DE therap* or DE session* or DE "parent mediated" or DE "parent delivered" or DE “family delivered” or DE "parent-delivered" or DE “family-delivered” or DE program or SU intervention or SU treatment or SU clinic or SU "parent training" or SU "parent program*" or SU "parent workshop" or SU "parent involvement" or SU "parents as teachers" or SU "home instruction" or SU "home intervention" or SU "home visits" or SU "home programs" or SU "family programs" or SU service* or SU counseling or SU therap* or SU session* or SU "parent mediated" or SU "parent delivered" or SU “family delivered” or SU "parent-delivered" or SU “family-delivered” or SU program or SU training or “train* for parent*” or “train* of parent*” or “parent* home interven*” or  “parent-mediated” or “parent mediated” or “parent* program*” or “parent* interven*” or “interven* with parent*” or “intervent* for parent*”)

**ProQuest Dissertations and Theses**

use the search string from Medline/ERIC

Database specific search terms

ERIC: SU DE KW

APA Psyinfo: SU DE

Academic search ultimate/Medline/Social work abstract: SU
